# Knockout of glucosidase II beta subunit inhibits growth and metastatic potential of lung cancer cells by inhibiting receptor tyrosine kinase activities

**DOI:** 10.1038/s41598-019-46701-y

**Published:** 2019-07-17

**Authors:** Worapong Khaodee, Suruk Udomsom, Phraepakaporn Kunnaja, Ratchada Cressey

**Affiliations:** 1 Division of Clinical Chemistry, Department of Medical Technology, Faculty of Associated Medical Sciences, Chiang Mai University, Chiang Mai, 50200 Thailand; 2 Cancer Research Unit of Associated Medical Sciences (AMS-CRU), Faculty of Associated Medical Sciences, Chiang Mai University, Chiang Mai, 50200 Thailand; 3 Biomedical Engineering Program, Faculty of Engineering, Chiang Mai University, Chiang Mai, 50200 Thailand; 4 Biomedical Engineering Center, Chiang Mai University, Chiang Mai, 50200 Thailand

**Keywords:** Targeted therapies, Cancer models

## Abstract

Glucosidase II (GluII) plays a major role in regulating post-translation modification of N-linked glycoproteins. We have previously reported that the expression of glucosidase II beta subunit (GluIIβ) was significantly increased in lung tumor tissues and its suppression triggers autophagy and/or apoptosis. Here, we investigated the role of GluIIβ in cell growth, metastatic potential, and receptor tyrosine kinases (RTKs) signaling activity in lung carcinoma cell lines. CRISPR-CAS9 technology was used to knockout the GluIIβ encoding gene (PRKSH) in lung carcinoma cells. GluIIβ knockout cells exhibited drastically slower growth rates in comparison to non-target transfected cells, particularly with lower concentrations of fetal bovine serum, indicating impairment of their ability to survive under nutritional deprivation. Cell migration and anchorage-independent growth, the fundamental components of cancer cell metastasis, were significantly decreased in GluIIβ knockout cells. Knockout of GluIIβ increased the sensitivity of lung cancer cells to cisplatin but reduced their sensitivity to gefitinib. Interestingly, knocking out of GluIIβ lowered overall RTK signaling activities to less than half of those in non-target transfected cells, which could represent a novel strategy for blocking multiple RTKs in tumor cells in an effort to improve lung cancer treatment.

## Introduction

N-linked glycosylation, a mechanism of protein processing that involves the attachment of a glycan to a protein, is crucial for determining the function, stability, and trafficking of glycoproteins. This posttranslational process is induced once newly synthesized protein enters into the endoplasmic reticulum (ER) and involves linking a carbohydrate molecule to an asparagine (N) amino acid residing within the specific consensus sequence NXS/T (reviewed in^1^). Glucosidase II beta subunit (GluIIβ) encoded from the *PRKCSH* gene functions as a beta subunit of glucosidase II, an enzyme involved in the regulation of N-linked glycosylation of multiple growth receptors. Only correctly folded proteins leave the ER to perform their activities as misfolded or improperly folded proteins are retained within the ER and subsequently degraded. The removal of a glucose molecule from N-linked glycoproteins by glucosidase II will permit their release from the ER, while the reversal of this process by UDP-glucose: glycoprotein glucosyltransferase 1 (UGT1) will cause these proteins to be withheld within the ER^2^. The balance between glucosidase II and UGT1 activity is fundamental to maintain the quality of the protein folding process within the ER. GluIIβ was reported to be frequently overexpressed in non-small cell lung carcinoma (NSCLC)^3^ and suppression of its expression and/or activity has been reported to dose dependently inactivated EGFR/RTK and PI3K/AKT signaling pathways^4^, causing autophagy^4,5^ and apoptosis^4,6^. The observations that GluIIβ suppression caused a decrease of EGFR/RTK and PI3K/AKT signaling activities lead to the hypothesis that tumor cells may rely on the activation of GluIIβ expression to help activate RTKs activities and advance their progression. This study investigated the impact of GluIIβ knockout on the growth behaviors, metastatic potential and RTKs signaling activities in lung cancer cell lines.

## Material and Methods

### Chemical

Antibodies to glucosidase II beta subunit and actin, were obtained from Santa Cruz Biotechnology, Inc. (Texas, USA). Horseradish peroxidase-conjugated anti-mouse immunoglobulin G (IgG) were from DakoCytomation (Denmark). Clarity™ ECL Western Blotting Substrate were obtained from Bio-Rad Laboratories (California, USA).

### Cell lines

A549 and H1299 cells were obtained from American Tissue Culture Collection (ATCC). A549 human lung carcinoma cells were maintained in DMEM. Human, p53-deficient cancer cell line H1299 was maintained in RPMI 1640. Both DMEM and RPMI were supplemented with 10% fetal bovine serum (FBS) (v/v), 100 units/ml penicillin and 100 µg/ml streptomycin (Gibco-Thermo Fisher Scientific, (Massachusetts, USA)).

### Knockout of GluIIβ using CRISPR/Cas9-mediated genome editing

A GluIIβ knockout A549 and H1299 lung cancer cell line were established by CRISPR/Cas9-mediated genome editing. Transfection was conducted according to the Santa Cruz Double Nickase transfection’s protocol. Briefly, about 2 × 10^5^ cells/well were seeded and cultured in a six well tissue culture plate overnight. 15 μl of (1.5 µg) of Glucosidase IIβ Double Nickase Plasmid (sc-404394-NIC, Santa Cruz Biotechnology, Texas, USA) or Control Double Nickase Plasmid (sc-437281, Santa Cruz Biotechnology, Texas, USA) diluted in incomplete media (DMEM for A549 and RPMI1640 for H1299) was mixed with 10 μl of UltraCruz® Transfection reagent (sc-395739, Santa Cruz Biotechnology, Texas, USA) and incubated for 45 minutes at room temperature. After replacing the cultured media with fresh antibiotic-free medium, the plasmid DNA/UltraCruz® transfection reagent complex was then added dropwise with gentle swirling into cultured cells. Seventy-two hours after transfection, cells were cultured in puromycin containing media for 3 weeks. Colonies of surviving cells were individually picked (or pooled together) and expanded into larger vessels before subjecting to further tests.

### Cell viability assay

Transfected cells (approximately 1 × 10^4^ cells) were seeded in 96-well plates at a density of 40–50% (total volume of 200 μl per well) and left overnight at 37 °C and 5% CO_2_. At each time point, 20 μL of 3–4,5 dimethyl thiazol 2,5 diphenyl tetrazolium bromide (MTT) solution (5 mg/mL) were added to each well. Incubation with MTT was terminated after 4 hours. The resulting violet formazan precipitate was solubilized by the addition of 100 µl dimethyl sulfoxide (DMSO) and the absorption was measured at 595 nm (Emax Plus microplate reader, Molecular Devices, California, USA).

### RT-PCR

Gene transcripts of GluIIβ in GluIIβ-knockout cells and non-target transfected cells were examined using SuperScript® III One-Step RT-PCR System with Platinum® Taq DNA Polymerase (Invitrogen, Thermo Fisher Scientific, USA) according to the user’s guide. The system uses a mixture of SuperScript® III Reverse Transcriptase and Platinum® Taq DNA polymerase to detect a wide range of RNA targets, from 200 bp to 4.5 kb.

Total RNA (1 μg) was reverse-transcribed at 55 °C for 30 minutes. The synthesized cDNA was then pre-denatured at 94 °C for 2 minutes and amplified for 40 cycles at 94 °C for 1 minute, 55 °C for 1 minute and 68 °C for 3 minutes using primers specific for GluIIβ (GluII Fw1: 5′-ATG GCG GCG GTA GCG GCA GT-3′ GluII Rv3: 5′-TTA TCG CAG GTG AAT ACT CCA ATC AGA TGC-3′). The PCR product was then analyzed using agarose gel electrophoresis.

### Western blot analysis

Treated cells were lysed in sodium dodecyl sulfate (SDS) lysis buffer [0.5 M Tris-HCl pH 6.8, 2% SDS (w/v) and 10% glycerol (v/v)] and heated at 95 °C for 10 minutes. The cell lysate was then centrifuged at 10,000 x g for 15 min at 4 °C, after which the supernatant was collected and the protein was determined using a BCA protein assay kit (Pierce Biotechnology, Illinois, USA). Cell lysate (total protein of 25–35 µg) from each treated condition was resolved on SDS polyacrylamide gels under reducing conditions and electrotransferred onto a PVDF membrane (Pall Corporation, New York, USA). After blocking with 5% non-fat milk in Tris buffer saline (TBS) containing 0.05% Tween-20 (TBS-T), the membrane was incubated with primary antibodies for 1 hour at room temperature. Bound antibodies were then detected with horseradish peroxidase (HRP)-conjugated goat anti-mouse IgGs for 1 hour. After extensive washing, immunoreactive protein was visualized with a chemiluminescence-based procedure using the Clarity ECL detection kit (Biorad Laboratories, California, USA) and x-ray film (Kodak, New York, USA)

### Anchorage independent growth assay

The anchorage independent growth of GluIIβ knockout cells was assessed according to Ke *et al*.^7^. In brief, a mixture of 25 μL pre-warmed (37 °C) 2x DMEM or 2x RPMI containing 20% FBS, 200 U/mL penicillin/streptomycin (Invitrogen), and 25 μL pre-warmed (56 °C) 0.8% agarose were plated onto each well of a 96-well microplate to serve as a pre-layer for the assay. Ten microliters of cell suspensions containing 1.5 × 10^3^ cells were mixed with 20 μL 2× DMEM and 30 μL 0.6% agarose and transferred to the 96-well microplate containing the solidified pre-layers. Semisolid feeder layers were then prepared by mixing 25 μL 2× DMEM and 25 μL 0.8% agarose and layered on top of the solidified cell layers. The cells were allowed to grow in the humidified 37 °C incubator with 5% CO_2_ for 1 to 2 weeks before cell proliferation and viability were scored using alamarBlue assay methods. Cell growth was measured using a Synergy™ 4 Multi-Detection Microplate Reader (BioTek, MA, USA), with excitation at 540 nm and emission at 590 nm.

### Cell migration assay

Migration assays were performed in Transwells (3428, Corning Inc., 8.0-μm pore size). After verification of GluIIβ knockout, 1.0 × 10^6^ cells in serum-free medium were added to the upper chamber of 6-well transwell plate. Media containing 20% FBS were added to the lower wells. The cells were allowed to invade for 16 hours (37 °C, 5% CO_2_ atmosphere), and the chambers were then washed with PBS. Those cells that did not invade through the membrane were removed. The migrated cells on the lower surface of the membrane were fixed with cold methanol, stained with 0.2% crystal violet and photographed under a light microscope. Afterward, membranes with migrated cells were extracted and soaked in 200 μl of DMSO and subjected to optical density measurement at 595 nm (Emax Plus microplate reader, Molecular Devices, California, USA) using DMSO as blank, in order to quantify migrated cells.

### Clonogenic assay

Cell sensitivity to cisplatin and gefitinib was assessed by colony-forming assay. Transfected cells were harvested and seeded in 60-mm dishes in triplicate (1000 cells/dish). 24 hours later, cells were exposed to 0.75 μg/ml cisplatin or 10 μg/mL gefitinib for 72 hours. After removing the drug, the cells were washed with PBS and then incubated in drug-free medium until colonies of control cells become evident (approximately 15 days later). Samples were stained with 1% crystal violet in methanol for 1 hour, and visible colonies were counted.

### Receptor tyrosine kinase phosphorylation profile

To investigate the activation/phosphorylation of RTKs, we used the Human RTK Phosphorylation Antibody Array Membrane (ab193662, Abcam). The human phospho-RTK antibody array is a nitrocellulose membrane with 71 different anti-RTK antibodies spotted in duplicate on it, including 4 positive and 3 negative controls, and 1 blank.

To conduct a proteome profiler array experiment, cell lysate was prepared from GluIIβ knockout cells and non-targeted transfected cells using Cell Lysis Buffer supplemented with Phosphatase Inhibitor and Protease Inhibitor Cocktail and stored at −80 °C until use. For each cell lysate, 200 μg of total protein (determined by the Pierce BCA Protein Assay (Fisher Scientific)) was diluted 1:5 with blocking buffer and placed onto each membrane and incubated overnight at 4 °C (16 hours). The antibody array membranes were washed and subsequently incubated with biotinylated anti-phosphotyrosine antibody overnight at 4 °C to detect phosphorylated tyrosines on activated receptors. After washing and incubation with HRP-streptavidin, the membranes were subjected to visualization with chemiluminescence-based detection method.

### Statistical analysis

Statistical analysis was performed using SPSS software version 15 (SPSS, Inc., Chicago, IL, USA). Comparison of different groups was performed with the Mann–Whitney U test, with P < 0.05 considered significant.

## Results

### Knockout of PRKCSH using CRISPR/Cas9-mediated genome editing increases cell doubling time

**Figure 1:**
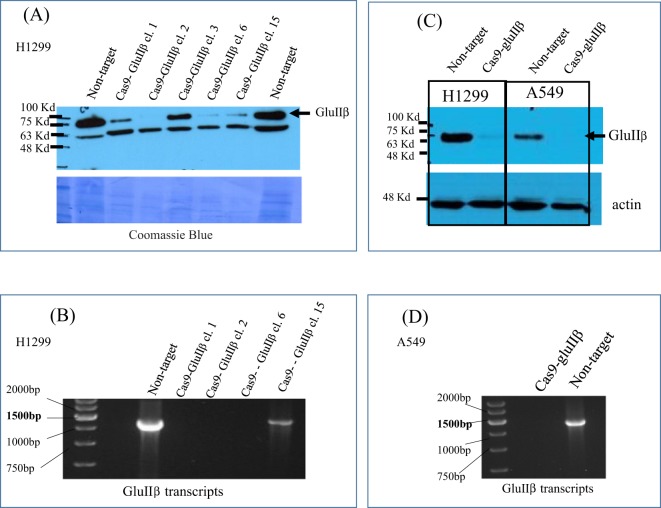
Representative results from Western blot analysis (**A**,**C**) and RT-PCR (**B**,**D**) verifying the suppression of GluIIβ expression in individual stable clones (**A**,**B**) and pooled stable clone (**C**,**D**) cells transfected with Cas-9-GluIIβ in comparison to non-target transfected cells. Full images of western blot results and RT-PCR results are provided in the supplemetary file.

To clarify the function of GluIIβ in lung cancer cells, we knockout PRKCSH, the GluIIβ encoding gene, in A549 and H1299 non-small cell lung cancer cells. Approximately three weeks after transfection, stable clones were picked and expanded. From the colony of cells that were picked, out of 5 surviving clones, clone number 2 had undetectable levels of GluIIβ transcript and protein (Fig. 1A,B), and was subjected for further analysis. For some experiments, stable clones were pooled together, and results of Western blot analysis and RT-PCR showed undetectable level of GluIIβ transcripts and protein in these pooled clones (Fig. 1C,D).

**Figure 2:**
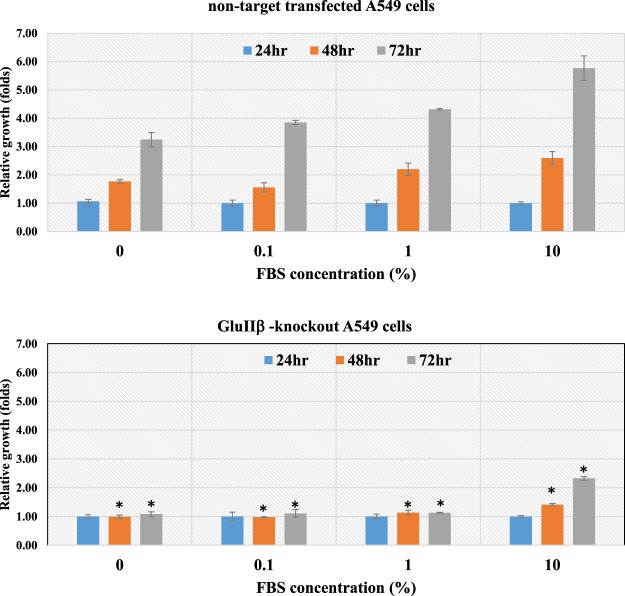
Growth behaviors of GluIIβ knockout cells under various concentrations of fetal bovine serum (FBS) compared to non-target transfected cells. After verification of GluIIβ suppression, transfected cells were seeded in 96-well plates and cultured in media supplemented various concentrations of FBS (0, 0.1, 1.0, 10% v/v) for 72 hours. Every 24 hours, cell multiplications were assesses by MTT assay. Bar graphs represent means and standard deviations (SD) of relative growth from three independent experiments in A549 cells. *Significant different from non-target transfected cells compared between cells cultured in the same concentration of FBS and at the same period of time (p < 0.05 by Mann Whitney U test).

After verification of GluIIβ suppression, transfected cells were cultured in media containing various concentration of FBS (0%, 0.1%, 1.0%, 10%) in order to investigate their growth behavior under conditions of growth factors deprivation. The growth rate of GluIIβ knockout cells was drastically lower than those of non-target transfected cells (Fig. 2). GluIIβ knockout A549 cells did not proliferate at all at FBS concentration below 10% FBS (0%, 0.1% and 1.0%), whereas non-target transfected cells continue to proliferate at these concentrations. Doubling times of cells cultured at different FBS concentrations were calculated using free software (http://www.doubling-time.com/compute_more.php) and are shown in Table 1. Knocking out of GluIIβ caused the doubling time of A549 cells cultured in 10% FBS containing media to increase from 18.9 ± 1.97 to 38.5 ± 3.42 hours. The impact of GluIIβ knockout was even more prominent in the absence of FBS as the doubling time increased from 29.3 ± 3.51 to 405 ± 7.75 hours (Table 1). Knockout of GluIIβ also caused a significant increase in the cell doubling time in H1299 cells (Table 1).

**Table 1:** Cellular doubling times of GluIIβ knockout cells and their control counterparts.

		Cellular Doubling Time, hours (mean ± SD)			
		0% FBS	0.1% FBS	1.0% FBS	10.0% FBS
A549	Non-target transfected cells	29.4 ± 3.51	22.3 ± 2.56	23.2 ± 1.95	18.9 ± 1.97
	GluIIβ knockout cells	405 ± 7.75*	322 ± 8.92*	286 ± 10.81*	38.5 ± 3.42*
H1299	Non-target transfected cells	86 ± 7.21	85 ± 8.11	71 ± 5.77	26 ± 2.47
	GluIIβ knockout cells	163 ± 6.23*	119 ± 5.51*	79 ± 4.39	30 ± 1.88

Data was represented in mean ± SD of cellular doubling time from three independent experiments. *Significant different from non-target transfected cells (p < 0.05 by Mann Whitney U test).

### Knockout of PRKCSH decreases the metastatic potential of lung cancer cell lines

**Figure 3:**
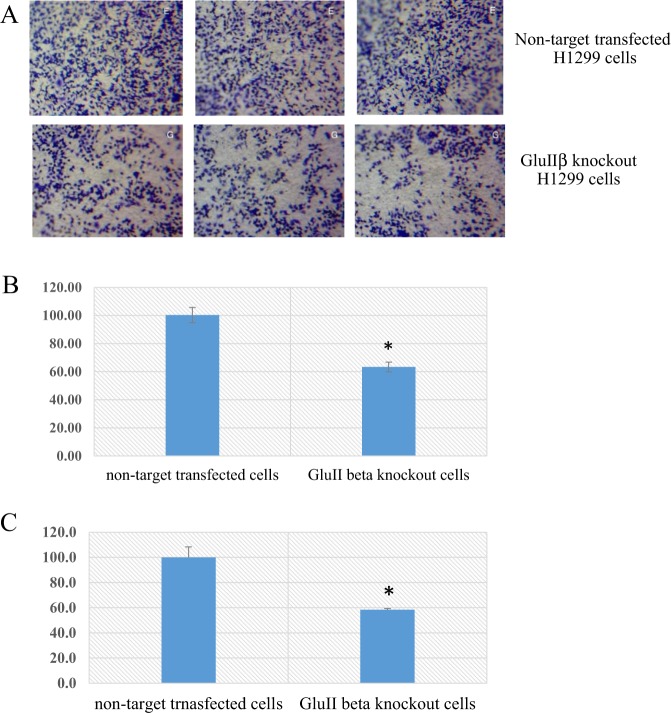
Representative images from transwell migration assay stained with crystal violet (**A**). Migrated cells were stained and then quantified by dissolved in DMSO and the optical density of the obtained solution was measured at 570 nm. OD_570nm_ from non-target transfected cells were calculated as 100% and relative percentage of GluIIβ knockout cells were calculated. Bar graphs represent means and standard deviations (SDs) of relative OD570nm compared to non-target control cells from three independent experiments in A549 (**B**) and H1299 (**C**). *Significant different from non-target transfected cells (p < 0.05 by Mann Whitney U test).

**Figure 4:**
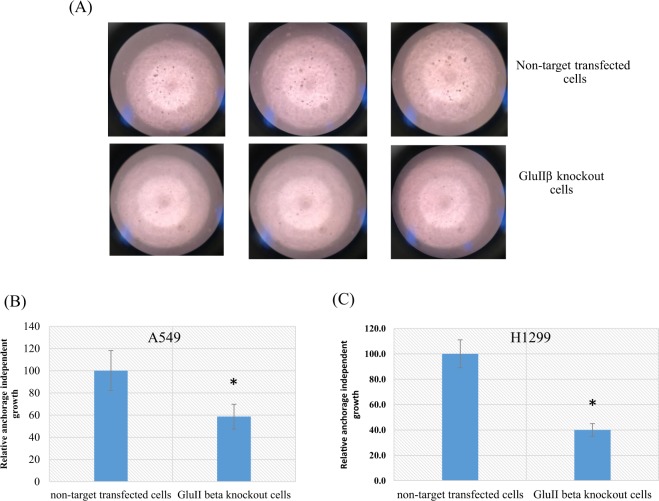
Representative images from anchorage-independent growth assay (**A**). Colonies formed in soft agar were quantified by staining with alamar Blue and the fluorescence intensities were measured with excitation wavelength of 540 nm and emission wavelength of 590 nm. Bar graphs represent means and standard deviations (SD) of relative fluorescence intensities compare to non-target transfected cells from three independent experiments in A549 (**B**) and H1299 (**C**). *Significant different from non-target transfected cells (p < 0.05 by Mann Whitney U test).

Cell migration and anchorage independent growth are fundamental components of tumor cell metastasis^8^. To assess the effect of GluIIβ on cell mobility, we examined the migration of GluIIβ knockout cells and their corresponding control using the transwell migration assay^9,10^. Figure 3A shows representative migrated cells stained with crystal violet examined after 16 hours of incubation. To obtain quantifiable data, migrated cells stained with crystal violet were dissolved in DMSO and the optical density plotted (Fig. 3B,C). It was found that knockout of GluIIβ suppressed cell migrating ability by 35 to 40%. Anchorage independent growth assays demonstrated that knockout of GluIIβ encoding gene also suppressed the colony-forming ability of lung cancer cells. Representative images of colonies formed in soft agar are shown in Fig. 4A. Colonies of cells were quantified by staining with alamar Blue and the fluorescence intensities were measured. Bar graphs showing the means and standard deviations (SDs) of relative fluorescence intensities compare to non-target control cells from three independent experiments in A549 (Fig. 4B) and H1299 (Fig. 4C) are shown. Knockout of GluIIβ suppressed anchorage-independent growth by 40 to 60%.

### Knockout of PRKCSH change sensitivity of lung cancer cells to chemotherapy

Given that knockout of GluIIβ reduced growth rate, we investigated whether knockout of GluIIβ enhanced the chemosensitivity of lung cancer cells to cisplatin or gefitinib, the two chemotherapeutic agents clinically used for lung cancer treatment.

**Figure 5:**
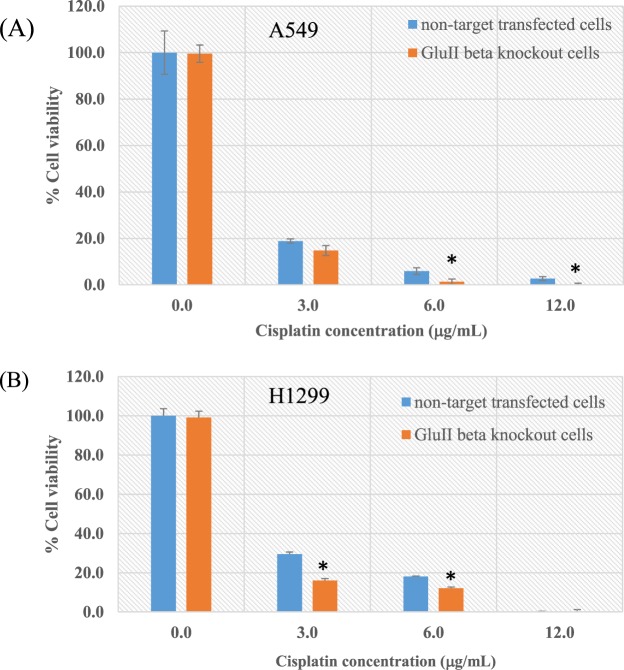
Knockout of GluIIβ sensitizes A549 (**A**) and H1299 (**B**) cells to cisplatin. After verification of GluIIβ suppression, transfected cells were seeded in 96-well plates and cultured in media supplemented various concentrations of cisplatin (0, 3, 6, 12 μg/mL) for 72 hours. After 72 hours, cell viabilities were assessed by MTT assay. Bar graphs are means and standard deviations (SD) of relative cell viabilities compared to non-treated cells from three independent experiments. *Significant different from non-target transfected cells (p < 0.05 by Mann Whitney U test).

**Figure 6:**
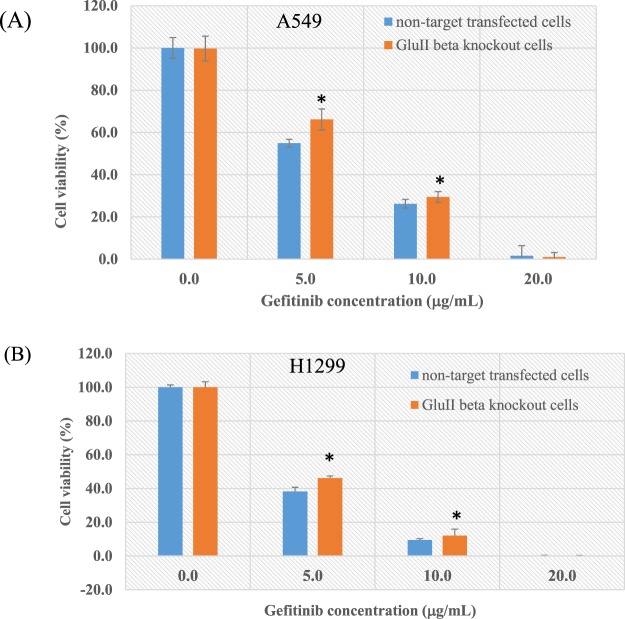
Knockout of GluIIβ reduces sensitivities of A549 (**A**) and H1299 (**B**) cells to gefitinib. After verification of GluIIβ suppression, transfected cells were seeded in 96-well plates and cultured in media supplemented various concentrations of gefinitib (0, 5, 10, 20 μg/mL) for 72 hours. After 72 hours, cell viabilities were assessed by MTT assay. Bar graphs are means and standard deviations (SD) of relative cell viabilities compared to non-treated cells from three independent experiments. *Significant different from non-target transfected cells (p < 0.05 by Mann Whitney U test).

**Figure 7:**
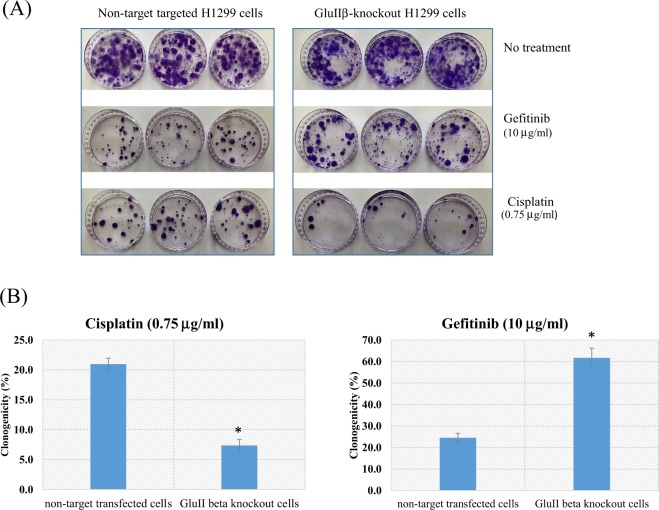
Knockout of GluIIβ decreases clonogenic ability of H1299 after treatment with cisplatin but increase after treatment with gefitinib. Representative images from clonogenicity testing after being exposed to cisplatin and gefitinib in H1299 cells stained with crystal violet (**A**). After verification of GluIIβ suppression, 500 of transfected cells were seeded in triplicated in 60 mm dish and cultured for overnight before treated with either cisplatin (0.75 μg/mL) or gefinitib (10 μg/mL) for 72 hours, or left untreated. After removing the drug, the cells were washed and continued cultured in drug-free medium for 2–3 weeks. Cells were stained with crystal violet and visible colonies were counted. Bar graphs represent means and standard deviations (SD) of relative number of colonies compared to those in non-treated cells from three independent experiments (7B). *Significant different from non-target transfected cells (p < 0.05 by Mann Whitney U test).

GluIIβ knockout cells and non-target transfected cells were treated with different concentrations of cisplatin and gefitinib for 72 hours. MTT assays revealed that all of the tested compounds reduced cell viabilities in a dose-dependent manner. Interestingly, while the growth suppression effect of cisplatin was enhanced in GluIIβ knockout cells both in A549 (Fig. 5A) and H1299 cells (Fig. 5B), sensitivity of knockout cells to gefitinib was decreased in comparison to non-target transfected cells (Fig. 6A,B). Results from clonogenicity assays demonstrated the same findings as from the MTT assay (Fig. 7A). Colonies of surviving cells after cisplatin treatment were about 20% in control cells compared to to 5% in GluIIβ knockout cells. In contrast, in response to gefitinib treatment, colonies of surviving cells were higher in GluIIβ knockout cells compared to control cells, 60% versus 25% (Fig. 7B).

### Knockout of PRKCSH decreased overall RTKs signaling activity

**Figure 8:**
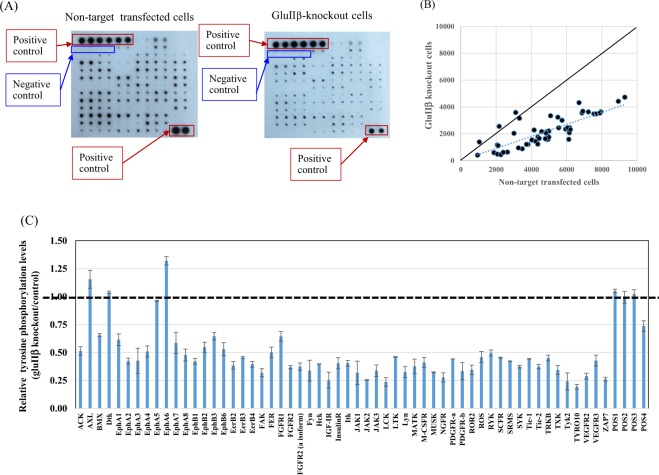
Analysis of RTK Phosphorylation levels. Image of RTK phosphorylation signals hybridized with cells lysate prepared from non-target transfected cells and GluIIβ knockout cells using Human RTK Phosphorylation Antibody Array Membrane (ab193662, Abcam) where 71 different anti-RTK antibodies have been spotted in duplicate, including 4 positive and 3 negative controls and 1 blank (**A**). Array signals were analyzed using “ImageJ software with the Protein Array Analyzer plugin16”. Values from duplicate spots were averaged and plotted (**B**), and the relative signal was calculated in GluIIβ knockout cells, compared to non-target transfected control cells (**C**).

Using Abcam’s Human RTK Phosphorylation Antibody Array Membrane, relative activities of 71 different human receptor tyrosine kinases (RTKs) were simultaneously determined by monitoring the changes in protein tyrosine phosphorylation. Knockout of GluIIβ reduced the majority of tyrosine phosphorylation levels (Fig. 8A,B). Signal levels were measured using “ImageJ software with the Protein Array Analyzer plugin16”. Values from duplicate spots were averaged, and the relative signal was calculated in GluIIβ knockout cells, compared to non-target transfected control cells. Tyrosine phosphorylation of 51 out of 71 receptor tyrosine kinases were reduced to less than half of those in non-target transfected cells cells (Fig. 8C).

## Discussion

We previously reported detecting increased expression of GlluIIß in a high proportion of lung carcinoma tissues^3^, and suppression of GlluIIß triggered cells to undergo autophagy and/or apoptosis^4–6^. In this study we have demonstrated that knockout of GlluIIß significantly decreased viability, migration, anchorage-independent growth and RTKs signaling activities of lung cancer cells.

GlluIIß is a beta subunit of an endoplasmic reticulum (ER) glucosidase II enzyme involved in the quality control of post-translation modification of N-linked glycoproteins. These groups of proteins comprise receptor tyrosine kinases (RTKs) that can initiate a number of downstream signaling cascades leading to cell growth, migration, differentiation, survival or apoptosis. RTKs are dysregulated by genetic alterations in some cancers and those cancers are often sensitive to tyrosine kinase inhibitors. Thus, RTKs are recognizable therapeutic targets for the treatment of various type of cancers. Two particular RTKs of high promise in oncology is the epidermal growth factor receptor (EGFR) and vascular endothelial growth factor receptors (VEGFRs)^11^. Two major classes of EGFR inhibitors have been successfully developed. The first class is monoclonal antibodies targeted against the extracellular domain of EGFR, e.g. cetuximab (Erbitux) and the second class is tyrosine kinase inhibitors (TKIs) e.g., gefitinib (Iressa) and erlotinib (Tarceva)^12^. The most common approaches to inhibit VEGFRs axis is blocking of VEGF ligands from binding to their receptors (i.e., using a monoclonal antibody or soluble receptor). Three FDA approved anti-angiogenic agents for cancer treatment are bevacizumab, ziv-aflibercept, and regorafenib^13^. Even though these RTKs inhibitors exhibit good clinical efficacy, patients frequently develops resistance within 1 to 2 years of treatment^14–17^. Accumulating evidences have demonstrated that cancer cells often develop redundant or compensatory survival pathways responsible for resistance through non-targeted glycoprotein RTKs^18–20^. Therefore, a therapeutic approach that interrupts multiple RTKs may provide improved clinical efficacy by blocking both the primary and the compensatory signaling pathways contributing towards tumor development and therapeutic resistance. Generally, RTKs are activated through the binding of its specific ligands. This allows a tyrosine residues in the cytoplasmic domains of dimeric receptors to be trans-phosphorylated by its partner receptor, transmitting a signal through the plasma membrane and initiating signal transduction pathways^21^. GlluIIß knockout cells showed universally lower levels of tyrosine phosphorylation in comparison to control cells, suggesting a broaden reduction of RTKs signaling activities. Following our observation that GlluIIß were frequently overexpressed in lung cancer cells^3^, we hypothesized that tumor cells may need high levels of GlluIIß to help promote their survival. It is possible that high levels of GlluIIß facilitates N-link glycosylation and thus over activates the RTK signaling pathways in tumor cells, which consequently helps promote their growth, migration and inhibition of apoptosis and/or autophagy. This could be the mechanism how knockout of GlluIIß in tumor cells leads to lower RTK activities that impairs their growth and survival, as shown in our study. Therefore, disrupting GluIIβ, i.e. with a selective inhibitor, represents a wider but targeted approach disrupting multiple RTKs signaling pathways and possibly more effective for cancer therapy.

Knockout of GluIIβ increased the sensitivity of lung cancer cells to cisplatin but, to our surprise, reduced their sensitivity to gefitinib. Gefitinib is a selective inhibitor of EGFR and reported to be effective in cancers with mutated and overactive EGFR^22,23^. The phosphorylation level of EGFR in our study was lower than the detection limit of the RTK Phosphorylation Antibody Array, thus EGFR phosphorylation was not one of the 51 suppressed RTKs identified in GluIIβ knockout cells. Nevertheless, we have previously demonstrated that suppression of GluIIβ activity decreased EGFR/RTK signaling activity by a flow cytometry-based method^4^. Thus, we hypothesizes that GluIIβ knockout cells may already possess lower levels of EGFR together with other RTKs activities and thus any additional blocking of EGFR by gefitinib does not significantly further impact their growth. This may explain why GluIIβ knockout cells became less sensitive to gefitinib in our study. The suppression of N-linked glycosylation by tunicamycin has been previously reported to markedly reduce RTKs signaling and radiosensitized U251 glioma and BXPC3 pancreatic adenocarcinoma cell lines (both of which became resistant to EGFR–targeted therapies)^24^. Therefore, reduced sensitivity of GluIIβ knockout cells to gefitinib may provide further evidence that suppression of GluIIβ reduces EGFR and overall RTKs activities.

RTKs are prime targets for cancer therapy in many types of cancer^25–27^. Since N-linked glycosylation is a critical step in the maturation of RTKs, its disruption has been proposed to be an effective strategy to target both primary and redundant RTK signaling^24,28^. However, normal cells may also rely on this pathway for their growth and development^29,30^. Nevertheless, suppression of GluIIβ may represent a novel approach to selectively inhibit N-linked glycosylation and thus specifically reduce RTKs activities in tumor cells. We have previously shown that expression levels of GluIIβ was barely detectable in normal lung tissues while significantly increased in cancer tissues^3^; therefore, blocking GluIIβ may represent a more targeted approach of blocking RTKs in tumor cells.

Autophagy activation during treatment with RTK inhibitors has been commonly observed^31,32^. Several signaling pathways triggered after activations of RTKs are known regulators of the autophagic process, i.e. mTOR^33^, thus RTKs inhibition can have a direct impact over autophagy regulation. Accordingly, suppression of GluIIβ activity either by a selective inhibitor or siRNA technology has been repeatedly reported to cause an induction of autophagy^4,5^, which may additionally be support by our finding that knock-out of GluIIβ down-regulated multiple RTK signaling activities leading to autophagy. Our previous study demonstrated that suppression of GluIIβ not only induced autophagy but also induced apoptosis, and the inhibition of autophagy could enhance the apoptosis inducing effect of GluIIβ suppression. Although GluIIβ suppression may represent a better approach by blocking multiple RTKs signaling pathways, without inhibition of autophagy this approach may also lead to autophagy and acquired resistance. Therefore, treatment with a GluIIβ inhibitor combined with the inhibition of autophagy represents a very effective approach to induce apoptotic cell death of cancer cells^4^.

In summary, our experiments show that inhibition of GluIIβ represents a novel strategy for blocking RTK signaling both within and across RTK families and signifies a targeted approach to disrupt signaling through multiple RTKs. Nevertheless, concomitant therapy with autophagy inhibitor in order to prevent acquire resistance is recommended.

## Supplementary information

Dataset 1
