# Supplementary material for: Knockout of glucosidase II beta subunit inhibits growth and metastatic potential of lung cancer cells by inhibiting receptor tyrosine kinase activities
Source: Sci Rep. 2019 Jul 17;9:10394. doi: 10.1038/s41598-019-46701-y (PMC6637200; doi:10.1038/s41598-019-46701-y)
Supplement: Supplementary file 1 — Dataset 1 [file 41598_2019_46701_MOESM1_ESM.pdf]

Knockout of glucosidase II beta subunit inhibits growth and metastatic potential of lung cancer cells by inhibiting receptor tyrosine kinase activities

Khaodee, Worapong[1]; Udomsom, Suruk[3,4]; Kunnaja, Phraepakaporn [1] and Cressey, Ratchada[*1,2]

[1]Division of Clinical Chemistry, Department of Medical Technology, Faculty of Associated Medical Sciences, Chiang Mai University, Chiang Mai 50200, Thailand

[2]Cancer Research Unit of Associated Medical Sciences (AMS-CRU), Faculty of Associated Medical Sciences, Chiang Mai University, Chiang Mai 50200, Thailand

[3]Biomedical Engineering Program, Faculty of Engineering, Chiang Mai University, Chiang Mai 50200, Thailand

[4]Biomedical Engineering Center, Chiang Mai University, Chiang Mai 50200, Thailand

*Correspondence to Ratchada.cr@cmu.ac.th

Fig 1

(A)

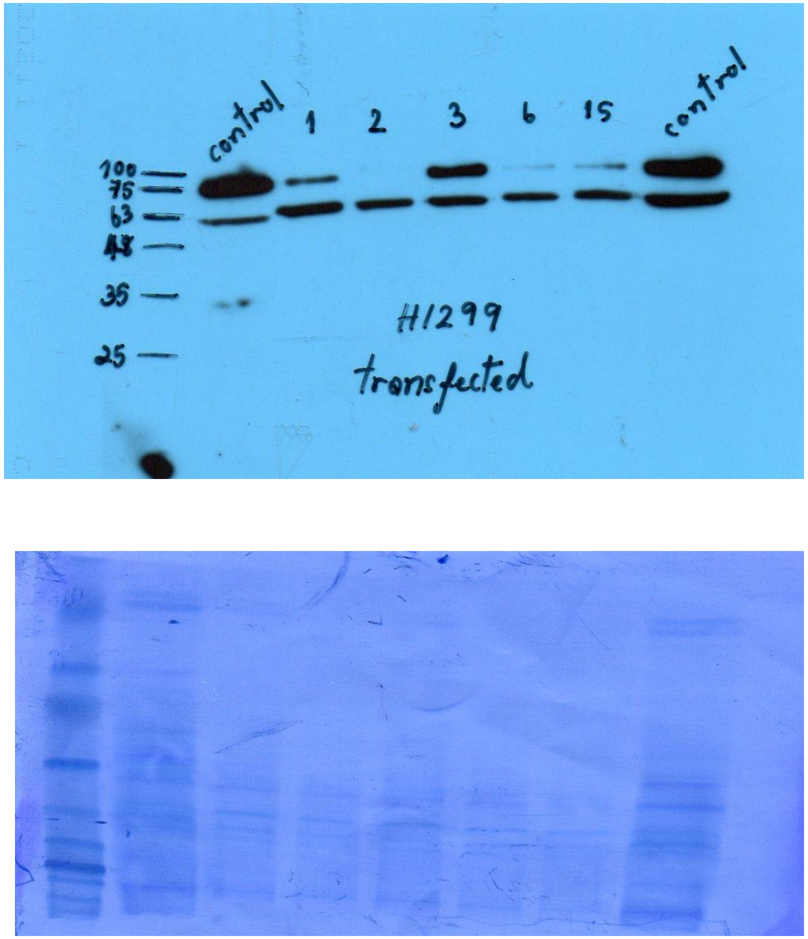

Coomassie Blue

Fig 1

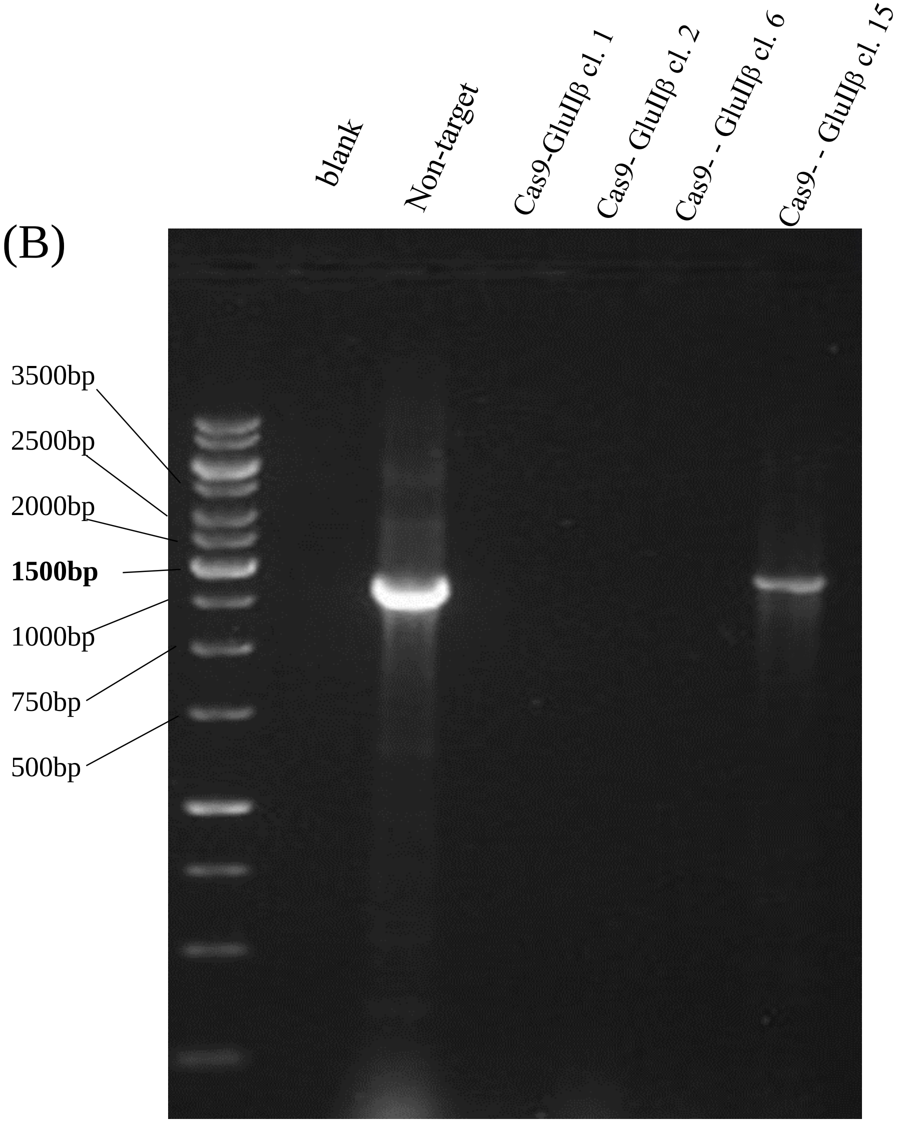

GluII transcripts

Fig 1

(C)

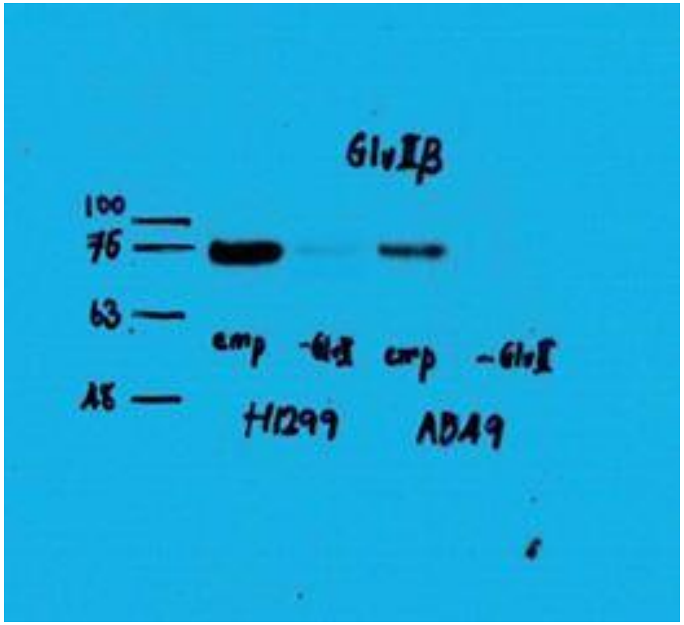

GluIIβ
100
75
63
45
emp
-GluII
emp
-GluII
H1299
A549

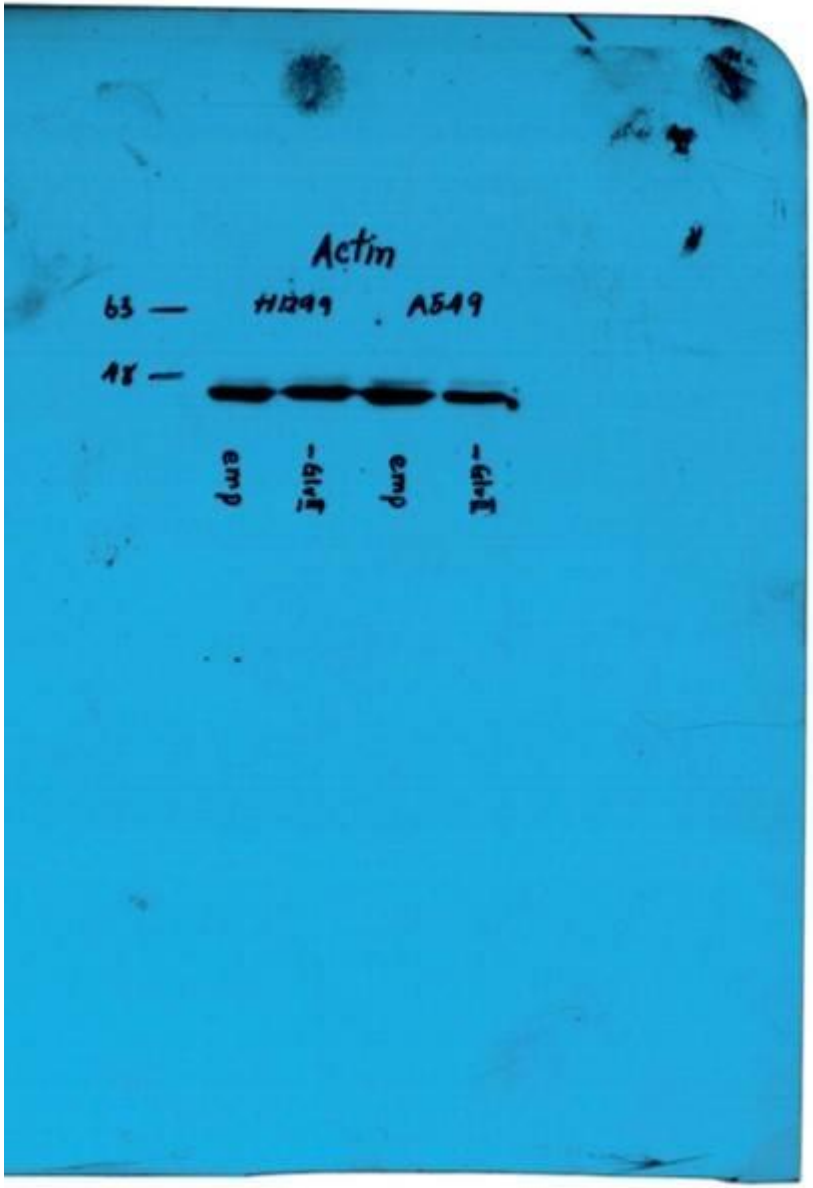

Actin
63
48
H1299
A549
emp
-GluII
emp
-GluII

Fig 1

(D)

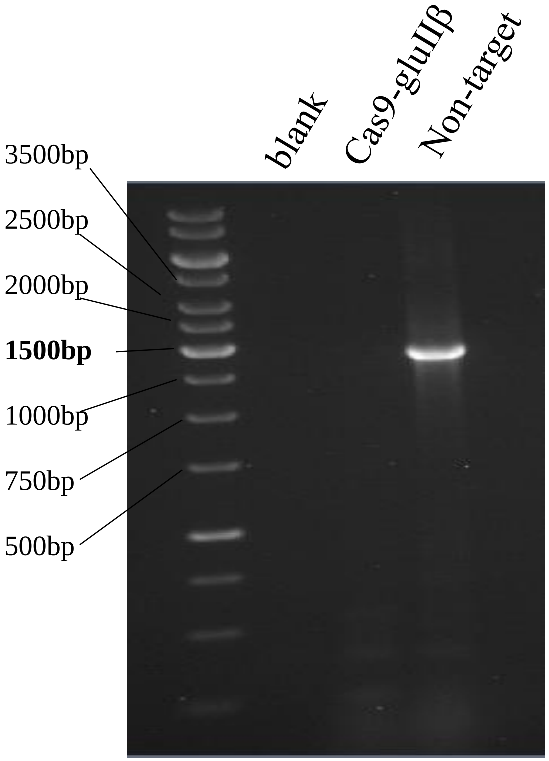

GluIIβ transcripts
